# Assessment of parental involvement in child health services for early childhood development and associated factors among children under 5 years old in Kolfe Keraniyo Sub City, Addis Ababa, Ethiopia: A cross-sectional study

**DOI:** 10.1097/MD.0000000000049860

**Published:** 2026-07-24

**Authors:** Mequanente Dagnaw, Meera Indracanti, Ellojita Rout, Zebiba Kedir, Suleyman Mohammed Arage

**Affiliations:** a Department of Epidemiology and Biostatistics, Institute of Public Health, University of Gondar, Gondar, Ethiopia; b Department of Medical Biotechnology, Institute of Biotechnology, University of Gondar, Gondar, Ethiopia; c Department of Medical Biotechnology, School of Allied and Healthcare Sciences, Malla Reddy University, Hyderabad, Telangana, India; d Department of Microbiology, Malla Reddy Medical College for Women, Malla Reddy Vishwavidyapeeth, Hyderabad, Telangana, India; e Department of Public Health, Ayer Tena Health Science and Business College, Addis Abeba, Ethiopia; f Department of Public Health, College of Medicine and Health Science, Werabe University, Worabe, Ethiopia; g Department of Health System and Policy, Institute of Public Health, College of Medicine and Health Science, University of Gondar, Gondar, Ethiopia.

**Keywords:** development, early childhood development, growth, health services, healthcare, nutrition, parental involvement, under 5 years old

## Abstract

Parents play a key role in shaping their children’s feelings of safety through supportive approaches that foster mental and social growth. In contrast, harsh, abusive, or erratic parenting can erode the bond between parent and child, which may result in antisocial behaviors and mental health challenges in children. Hence, the importance of parents in delivering healthcare, nutrition, hygiene, and emotional support is vital for their growth and development. This study aimed to evaluate the participation of parents in child health (CH) services for early childhood development and the related factors for children below 5 years old in the Kolfe Karaniyo Sub City of Addis Ababa, Ethiopia, in 2024. A community-based cross-sectional study was conducted among 423 parents selected using systematic random sampling from 3 woredas. Data were collected through interviewer-administered questionnaires. Data entry was done using EpiData 3.1 and analyzed with SPSS version 27. Descriptive statistics were presented as frequencies and percentages. Bi-variable logistic regression (*P* < .25) was used to identify candidate variables, which were then included in multivariable logistic regression. Statistical significance was set at *P* < .05. In total, 59.9% (95% confidence interval [CI]: 55, 64.3) of parents exhibited a high level of engagement in CH services. This research uncovered several factors that were significantly linked to parental participation in these services. These factors include: the mother’s education level (primary and above): adjusted odds ratio (AOR) = 2.82, 95% CI: (1.73, 3.61), the mother’s employment status (housewife): AOR = 2.08, 95% CI: (1.24, 2.97), monthly income (high): AOR = 3.46, 95% CI: (2.95, 6.79), perception of healthcare affordability: AOR = 3.51, 95% CI: (2.19, 4.97), and a strong understanding of childhood developmental milestones: AOR = 2.33, 95% CI: (1.49, 3.64). These factors were identified as having significant correlations with parental involvement in CH services. The results of this research indicate a moderate degree of parental engagement in CH services when compared to other studies. Nonetheless, it is crucial to keep advocating for and improving parental participation in CH services to enhance outcomes.

## 1. Introduction

Early childhood development (ECD) encompasses the cognitive, physical, language, motor, and socio-emotional growth of children from birth to 8 years of age and is a critical foundation for lifelong health and well-being.^[1,2]^ The first 5 years of life are particularly sensitive, as rapid brain development during this period is strongly influenced by environmental conditions, nutrition, stimulation, and caregiving practices.^[3,4]^ Essential child health (CH) services including immunization, nutrition, sanitation, and access to safe water play a central role in supporting optimal development.^[5]^ Within this context, parents serve as primary caregivers, providing nurturing care that includes responsive interaction, emotional support, early learning opportunities, and a safe and stable environment.^[1,6]^

Parental involvement (PI) is a key determinant of CH and developmental outcomes. Positive parenting practices promote physical growth, cognitive development, and emotional well-being, whereas inconsistent or harsh caregiving can negatively affect children’s behavioral and mental health.^[7,8]^ In addition to mothers, fathers play a significant yet often underrecognized role in ECD. Evidence suggests that active paternal involvement contributes to improved cognitive outcomes, reduced behavioral problems, and enhanced psychosocial development.^[9,10]^ However, parental engagement is influenced by multiple factors, including sociodemographic characteristics, mental health, cultural norms, and access to health information and services.^[11]^

Globally, a substantial proportion of children fail to reach their developmental potential due to preventable risk factors such as poverty, malnutrition, inadequate stimulation, and limited access to healthcare.^[12]^ Sub-Saharan Africa bears a disproportionate burden, with high rates of child mortality and undernutrition.^[13]^ In Ethiopia, child malnutrition remains a major public health concern, with significant proportions of children experiencing stunting, wasting, and underweight.^[14]^ These challenges are closely linked to inadequate PI in CH services, feeding practices, and early stimulation, all of which are essential components of nurturing care.^[6,15]^

In the Ethiopian context, traditional gender roles often assign childcare responsibilities primarily to mothers, while fathers are expected to focus on economic provision. This division can limit comprehensive PI in CH and development.^[16]^ Furthermore, cultural beliefs and socioeconomic barriers may affect parents’ utilization of CH services and their engagement in development-promoting practices.^[11]^ Despite growing recognition of the importance of both parents in ECD, fathers remain underrepresented in research, and evidence on the combined role of parents in supporting ECD is limited.^[9]^

Although previous studies have highlighted factors influencing CH and development, there is a lack of evidence quantifying the role of PI in ECD in Ethiopia, particularly at the community level. Moreover, no study has specifically examined this issue in Kolfe Keraniyo Sub City, Addis Ababa. Therefore, this study aims to assess the role of parents in providing CH services for ECD among children under 5 years of age in Kolfe Keraniyo Sub City, Addis Ababa, Ethiopia, in 2024.

## 2. Materials and methods

### 2.1. Study area

Kolfe Keraniyo Sub City was one of Addis Ababa, Ethiopia’s 11 sub-cities. It had a population of 4,06,991 and 99,266 homes. The sub-city’s population was diversified, with various socioeconomic backgrounds. Kolfe Keraniyo’s health infrastructure is relatively advanced when compared to other parts of Addis Ababa, Ethiopia. The sub-city had both public and private health services, including a government hospital, 7 health centers, 5 private primary hospitals, and 1 private general hospital. The health bureau collaborates with the community, ECD (Early Childhood Development) program workers, and health extension workers to offer primary health care. Data for the study were gathered from the Kolfe Keraniyo Sub City Health Office.

### 2.2. Study design and period

A community-based cross-sectional quantitative study was conducted from August 1 to 30, 2024, using a structured interviewer-administered questionnaire for data collection.

### 2.3. Population

#### 2.3.1. Source population

The source population for this study included all parents of children aged 0 to 59 months (under 5 years) residing in Kolfe Keraniyo Sub City, Addis Ababa, Ethiopia, in 2024. This constituted the population from which the study participants were selected.

#### 2.3.2. Study population

The study populations were randomly selected parents who had children under 5 years old living in selected woredas of Kolfe Keraniyo Sub City, Addis Ababa, Ethiopia in 2024.

### 2.4. Inclusion and exclusion criteria

#### 2.4.1. Inclusion criteria

Parents (mothers and/or fathers) or primary caregivers of children aged 0 to 59 months residing in Kolfe Keraniyo Sub City, Addis Ababa, during the study period were included.

#### 2.4.2. Exclusion criteria

Parents (mothers, fathers, or primary caregivers) who were critically ill or unable to participate in an interview due to communication difficulties during the data collection period were excluded.

### 2.5. Sample size and sampling procedure

#### 2.5.1. Sample size determination

To calculate the sample size, the study used the following formula:


$$n=Z^{2}\times p(1-p)/d^{2},$$

where n = the required sample size, *Z* = the standard normal deviation, which is 1.96 for a 95% confidence level, *p* = the estimated proportion of the target population, which is usually set at 0.5 when the true proportion is unknown, *d* = the desired level of precision, which is usually set at 0.05 (or 5%).

Given the information provided: Population size (N) = 99,266 households, Confidence level = 95%, Margin of error (*d*) = 5% (0.05).

Plugging in the values:


$$\begin{array}{c} n={\left(1.96\right)}^{2}\times 0.5(1-0.5)/{\left(0.05\right)}^{2} \end{array}$$

$$\begin{array}{c} n=3.8416\times 0.25/0.0025 \end{array}$$

$$\begin{array}{c} n=384.16. \end{array}$$

Therefore, the recommended sample size for the study was 385 parents in the Kolfe Keraniyo Sub City of Addis Ababa, Ethiopia. Using the above formula, the calculated sample size was approximately 384 parents. However, to account for potential nonresponses and incomplete data, a total of 423 parents were targeted for data collection.

#### 2.5.2. Sampling technique and procedure

A simple random sampling technique was used to select 3 woredas (30%) from the total of 11 woredas in Kolfe Keraniyo Sub City. The total number of households in the selected woredas was 21,794 (Woreda 02 = 6230; Woreda 03 = 8120; Woreda 11 = 7444). Proportional allocation was applied to distribute the total sample size (n = 423) to each selected woreda based on the number of households using the formula: (number of households in each woreda ÷ 21,794) × 423. Accordingly, the allocated sample sizes were 121 for Woreda 02, 158 for Woreda 03, and 144 for Woreda 11.

Within each selected woreda, households were selected using systematic random sampling. The sampling interval (*k*) was determined by dividing the total number of households by the required sample size (*k* = N/n = 21,794/423 ≈ 52). The first household was selected randomly using a lottery method, and subsequently, every 52nd household was included.

In households with more than 1 eligible participant, 1 parent (mother or father) or primary caregiver of a child aged 0 to 59 months was selected randomly. Households without eligible participants were skipped, and the next immediate household was considered. Data collectors continued visiting households until the allocated sample size for each woreda was achieved.

### 2.6. Study variable

#### 2.6.1. Dependent variable

Parents’ involvement in CH services.

#### 2.6.2. Independent variables

Sociodemographic questions: Child’s age, child’s sex, father’s education, father’s occupation, mother’s education, mother’s occupation, number of children, and monthly income.
Access to healthcare services: It was measured using indicators such as distance to the nearest health facility, length of appointment (waiting time), satisfaction with healthcare services, and perceived adequacy of resources, reflecting the dimensions of accessibility, availability, accommodation, and acceptability.^[17,18]^
Parental knowledge: Knowledge of PI refers to parents’ understanding of engaging in activities that promote a child’s development, including responsive caregiving, early learning stimulation, and awareness of age-appropriate developmental milestones.^[1,6,19]^

### 2.7. Operational definitions of terms

- Good parental involvement: To assess the level of good PI on the child’s health development, 11 questions were asked, and if the parents’ score was above the mean score of the parent involvement questions.^[20,21]^
- Access to healthcare services: It was measured using indicators such as distance to the nearest health facility, length of appointment (waiting time), satisfaction with healthcare services, and perceived adequacy of resources, reflecting the dimensions of accessibility, availability, accommodation, and acceptability.^[17,18]^
- Parental knowledge on ECD milestone: Thirteen items related to parental knowledge on ECD were asked; after summing up those items, the participants’ knowledge levels were categorized as good if they scored above the mean score. Participants who obtained a score below the mean for each developmental domain were considered to have poor knowledge of a parental knowledge on ECD milestone.^[22]^

### 2.8. Data collection tool and procedure

#### 2.8.1. Data collection

A structured interviewer-administered questionnaire was used to collect data from each participant, covering both independent and dependent variables. The independent variables included sociodemographic characteristics of the mother, father, and child, as well as household economic status. Specifically, parental sociodemographic information such as age, educational level, and occupation was collected, while the child’s age and sex were recorded. Household monthly income was used to assess economic status. Access to healthcare services was evaluated in terms of availability and accessibility, including distance to health facilities, waiting time, service satisfaction, and resource adequacy. In addition, parental knowledge and PI defined as engagement in activities that support child development and awareness of early childhood developmental milestones were assessed.

The dependent variable was measured using 11 items adapted from previously published literature. The questionnaire was pretested on 5% of the sample outside the study area to ensure clarity and consistency. The reliability of the tool was assessed using Cronbach α, which demonstrated acceptable internal consistency (α ≥ 0.70). Necessary modifications were made based on the pretest findings before actual data collection.^[20,23]^

A scoring system was applied based on responses to 11 items assessing PI by converted Likert scale to binary. Each “agree” response was assigned a score of 1, while each “disagree” response was assigned a score of 0. The total score for each participant was calculated by summing the item responses. The mean score of the study sample was computed and used as the cutoff point for classification. Participants who scored above the sample mean were categorized as having “good involvement” (coded as 1), whereas those scoring below the mean were categorized as having “poor involvement” (coded as 0). This classification approach was adapted from previous studies conducted in similar settings; however, the use of a sample-based mean as a cutoff may limit external comparability across studies.^[10]^

#### 2.8.2. Data collection procedures

Data were collected using a structured interviewer-administered questionnaire. Study participants were approached and invited to participate voluntarily after a clear explanation of the study objectives during data collection. A total of 3 trained health extension workers served as data collectors, and 1 supervisor oversaw the data collection process. The questionnaire was pretested on 5% of the sample in a similar setting outside the study area to assess clarity, comprehension, and consistency. Based on the pretest findings, necessary modifications were made to improve the wording and flow of the questions.

Interviews were conducted at participants’ homes to ensure privacy and convenience. The average duration of each interview was approximately 20 to 30 minutes. Continuous supervision and daily checks were carried out to ensure data quality and completeness throughout the data collection period.

### 2.9. Data quality control

To assure data quality, clear and uniform data gathering processes were implemented. Prior to data collection, the questionnaire was pretested with 5% of the sample from a similar group who were not part of the primary study. The purpose of pretesting the questionnaire was to ensure high data precision and accuracy. The questionnaire was adjusted in accordance with the pretest results.

The lead investigator provided 2 days of rigorous instruction to data collectors. The data collectors were well-trained persons drawn from the community and Health Extension Workers. The training agenda includes study objectives, sampling processes, field operations (random household selection, introduction, and systematic data collection), questionnaire evaluation, guidelines for administering structured questionnaires, and interview techniques.

To ensure data quality, a systematic questionnaire was used to collect information. The questionnaire was created in English and translated into the local language (Amharic); its consistency was then verified by another person who speaks and understands both languages. The questionnaire was back translated into English to ensure conceptual equivalence. Variables in the questionnaire were developed from several questionnaires used for assessing similar studies.

### 2.10. Data processing and analysis

Data were entered into EpiData version 3.1 (The EpiData Association) and exported to SPSS version 27 (IBM Corporation) for analysis. Data cleaning was performed to check for completeness and consistency, and incomplete or inconsistent records were excluded. Descriptive statistics, including frequencies and percentages, were computed to summarize the characteristics of the study participants.

Bivariable logistic regression analysis was conducted to assess the association between each independent variable and the outcome variable. Variables with a *P*-value <.25 in the bivariable analysis were included in the multivariable logistic regression model to identify independent predictors. In the multivariable analysis, adjusted odds ratios (AORs) with 95% confidence intervals (CIs) were used to determine the strength and direction of associations, and statistical significance was declared at *P*-value <.05.

Model fitness was assessed using the Hosmer-Lemeshow goodness-of-fit test, and multicollinearity among independent variables was checked using variance inflation factor, with acceptable values considered to be <10.

## 3. Results

### 3.1. Sociodemographic characteristics

The study comprised 409 parents (father or mother) with children under the age of 5, resulting in a 96.7% response rate. In terms of age, 145 (35.5%) fathers were over 40 years old, whereas 272 (66.5%) women were under the age of 30. In terms of education and occupation, the majority of dads, 179 (43.8%), and 169 (41.3%), completed their primary education, while 172 (42.1%) of fathers and 239 (58.4%) of mothers worked as merchants and housewives. More than half (58.4%) of parents received information from a health care facility, while more than half (57.7%) of households had a low monthly income (Table 1).

**Table 1:** Sociodemographic characteristics of parents having children under 5 years old at Kolfe Karaniyo Sub City, Addis Ababa, Ethiopia 2024.

Variables	Category	Frequency	Percent
Child’s age	≤11 mo	64	15.6
	11–24 mo	160	39.1
	≥24 mo	185	45.2
Child’s sex	Male	170	41.6
	Female	239	58.4
Father’s age	25–29 yr	55	13.4
	30–34 yr	90	22.0
	35–39 yr	119	29.1
	Above 40 yr	145	35.5
	25–29 yr	55	13.4
Mother’s age	≤30 yr	272	66.5
	>30 yr	137	33.5
Number of children in the house	≤5 yr	346	84.6
	>5 yr	63	15.4
The father’s level of education	No formal education	86	21.0
	Primary	179	43.8
	Secondary and above	144	35.2
The mother’s level of education	No formal education	132	32.3
	Primary	169	41.3
	Secondary and above	108	26.4
Father’s occupation	Government employee	42	10.3
	Private employee	105	25.7
	Merchants	172	42.1
	Daily laborers	82	20.0
	Others	8	2.0
Mothers’ occupation	Government employee	28	6.8
	Housewife	239	58.4
	Private employee	97	23.7
	Merchants	34	8.3
	Daily laborers	11	2.7
How much is your monthly income	Low (<6273.84 birr/mo)	236	57.7
	High (≥6273.84 birr/mo)	173	42.3
Number of people in the household	<5	272	66.5
	≥5	137	33.5
Source of information about child health services	From a health facility	239	58.4
	Social media	100	24.4
	Friend and family	70	17.1

### 3.2. Parent involvement in child health services

**Figure 1.**
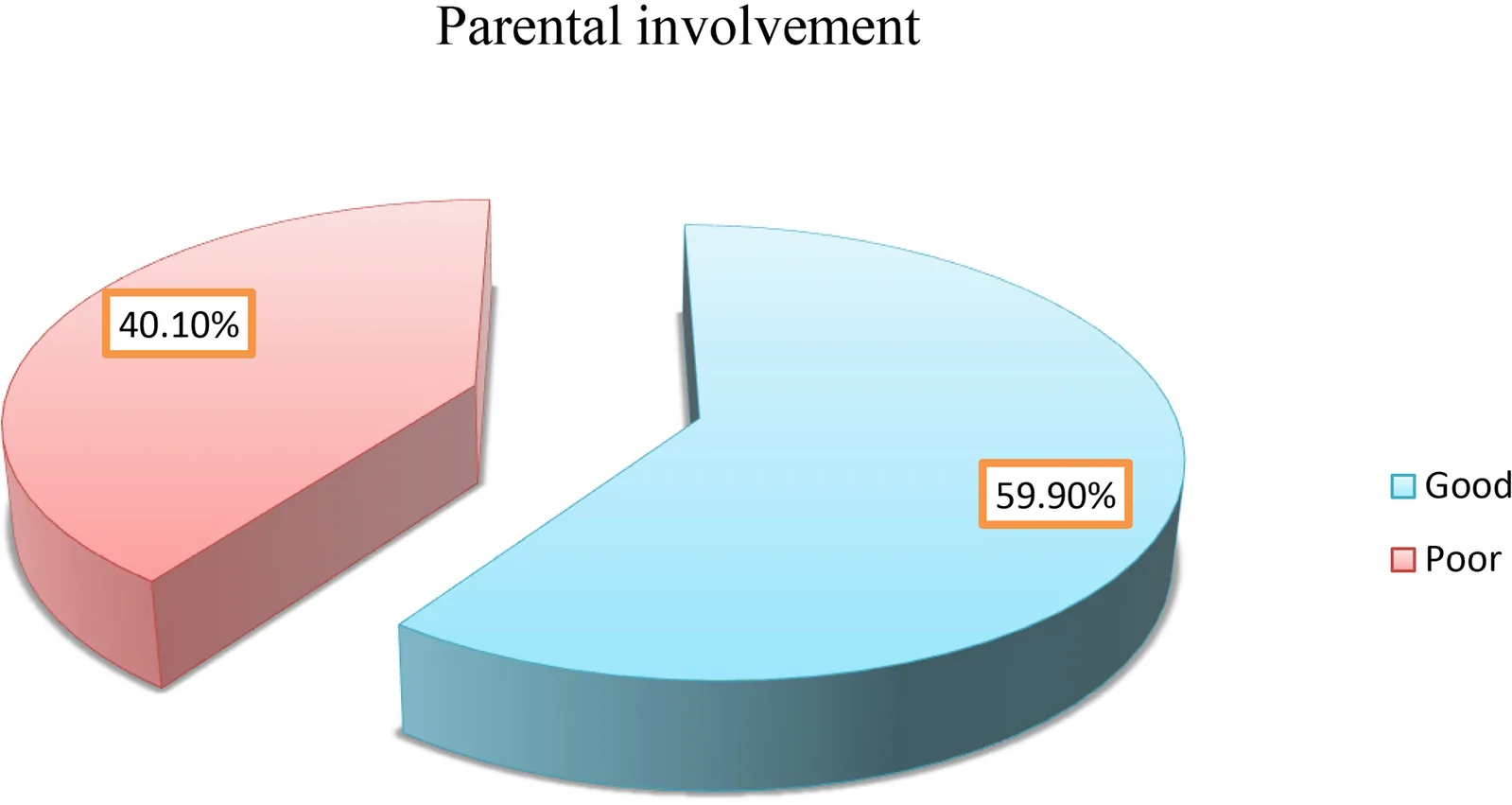
Parent involvement in child health service among parents having children under 5 years old at Kolfe Karaniyo Sub City, Addis Ababa, Ethiopia, 2024.

Regarding parental involvement status, 245 (59.9%, 95% CI: (55, 64.3) of the parents had good involvement in child health services, scoring above the mean score of 70.5%, whereas 164 (40.1%) had poor involvement (Fig. 1).

### 3.3. Healthcare services related factors

**Figure 2.**
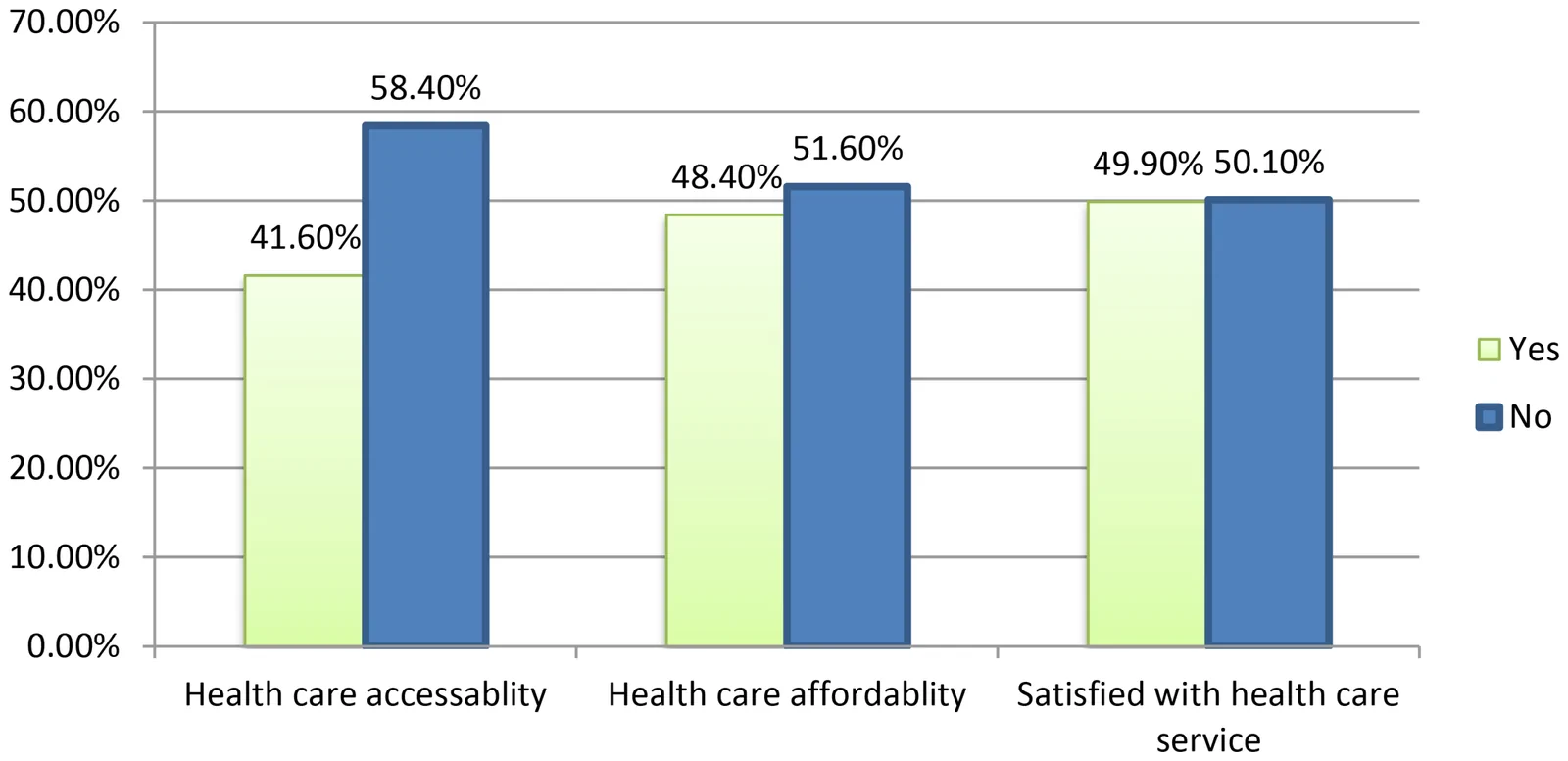
Healthcare services related factors among parents having children under 5 years old at Kolfe Karaniyo Sub City, Addis Ababa, Ethiopia, 2024.

Concerning health care service, 170 (41.6%) and 198 (48.8%) of the participants responded that health care service was accessible and affordable, respectively, while almost half (49.9%) of them were satisfied with health care service (Fig. 2).

### 3.4. Parental knowledge of child’s childhood developmental milestones and child health services

**Figure 3.**
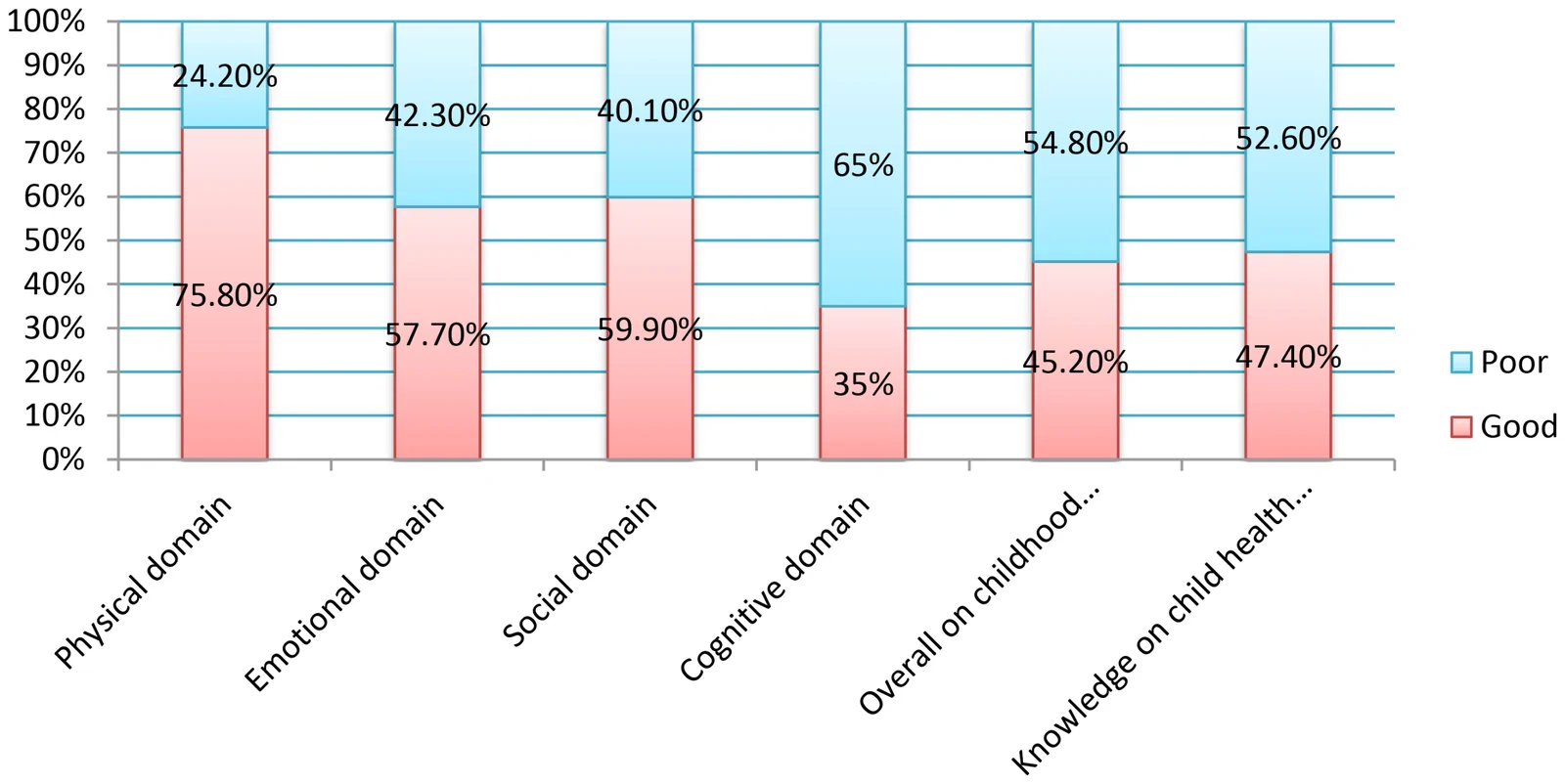
Parental knowledge on childhood developmental milestones and child health service among parents having children under 5 years old at Kolfe Karaniyo Sub City, Addis Ababa, Ethiopia, 2024.

In terms of parental knowledge of child developmental milestones, parents were more knowledgeable in the physical domain (310, 75.8%), followed by the social domain (245, 59.9%), but relatively low in the emotional domain (236, 57.7%) and the cognitive domain (143, 35%). Overall, 185 (45.2%) of parents had good understanding of childhood developmental milestones, and 194 (47.4%) had strong knowledge of CH services (Fig. 3).

### 3.5. Factors associated with parent involvement in child health services

In the final model (multivariable), mothers’ level of education, occupation, monthly income, health care service affordability, and understanding of developmental milestones were statistically significant with parental engagement (*P*-value <.05). When compared to moms with no formal education, the odds of parental participation in CH services were 2.82 and 1.76 times higher in those with primary and secondary or above educational levels, with AOR = 2.82, 95% CI: (1.73, 3.61) and AOR = 1.76, 95% CI: (1.12, 2.14), respectively. Housewives were 2.08 times more likely to participate in CH services than merchants and day laborers (AOR = 2.08, 95% CI: (1.24, 2.97). The odds of PI in CH care were 3.46 times higher among those with high income, AOR = 3.46, 95% CI: (2.95, 6.79).

Furthermore, individuals who perceived health care as affordable were 3.51 times more likely to be involved in child health services than those who did not (AOR = 3.51, 95% CI: (2.19, 4.97). Furthermore, odd parent involvement in CH services was 2.33 times higher among those who had good knowledge on childhood developmental milestones when compared to those who had poor knowledge, AOR = 2.33, 95% CI: (1.49, 3.64) (Table 2).

**Table 2:** Factors associated with parent involvement in child health services among parents having children under 5 years old at Kolfe Karaniyo Sub City, Addis Ababa, Ethiopia 2024.

Variable	Category	Parental involvement		COR	*P*-value	AOR 95% CI	*P*-value
		Good	Poor				
Child’s age	≤11 mo	43	21	1.25 (0.68, 2.27)	.472	1.12 (0.47, 1.13)	.342
	11–24 mo	87	73	0.73 (0.47, 1.12)	.144	0.48 (0.32, 1.09)	.121
	≥24 mo	115	70	1		1	
Father’s age	<30 yr	28	27	0.97 (0.52, 1.80)	.918	0.67 (0.41, 1.23)	.724
	30–39 yr	142	67	1.98 (1.28, 3.06)	.002	1.82 (0.98, 2.11)	.052
	≥40 yr	75	70	1		1	
Mother’s age	≤30 yr	157	115	1		1	
	>30 yr	88	49	1.32 (0.86, 2.01)	.205	1.14 (0.61, 1.91)	.142
Number of children in the house	≤5 yr	201	145	0.60 (0.34, 1.07)	.082	0.43 (0.24, 1.04)	0.68
	>5 yr	44	19	1		1	
Father’s level of education	No formal education	64	22	1		1	
	Primary	87	92	0.33 (0.19, 0.7)	.000	0.22 (0.09, 1.08)	.054
	Secondary and above	94	50	0.65 (0.36, 1.17)	.149	0.54 (0.28, 2.01)	.132
Mother’s level of education	No formal education	59	73	1		1	
	Primary	120	49	3.03 (1.88, 4.89)	.000	2.82 (1.73, 3.61)	.002
	Secondary and above	66	42	1.94 (1.16, 3.26)	.012	1.76 (1.12, 2.14)	.030
Father’s occupation	Government employee	27	15	2.25 (1.06, 4.79)	.035	2.13 (0.87, 2.28)	.074
	Private employee	88	17	6.47 (3.33, 12.58)	.000	4.23 (0.97, 7.83)	.053
	Merchants	90	82	1.37 (0.82, 2.29)	.226	1.21 (0.62, 1.97)	.216
	Daily laborers and others	40	50	1		1	
Mothers occupation	House wife	148	59	2.14 (1.33, 3.45)	.002	2.08 (1.24, 2.97)	.047
	Private employee	36	53	0.58 (0.33, 1.06)	.057	0.71 (0.49, 1.02)	.052
	Merchants and daily laborers	61	52	1		1	
Monthly income	Low	104	132	1		1	
	High	141	32	5.59 (3.52, 8.88)	.000	3.46 (2.95, 6.79)	.0001
Source of information about child health services	From a health facility	143	96	4.30 (2.37, 7.80)	.000	2.23 (0.86, 4.37)	.061
	Social media	84	16	15.17 (7.11, 32.36)	.000	6.24 (0.97, 4.68)	.063
	Friend and family	18	52	1		1	
Satisfied with the health care service	No	130	75	1		1	
	Yes	115	89	0.75 (0.50, 1.11)	.147	0.59 (0.41, 1.08)	.143
Health care affordability	Not affordable	97	114	1		1	
	Affordable	148	50	3.48 (2.29, 5.29)	.000	3.51 (2.19, 4.97)	<.0001
Knowledge of developmental milestones	Poor knowledge	113	111	1		1	
	Good knowledge	132	53	2.45 (1.62, 3.70)	.000	2.33 (1.49, 3.64)	<.0001

## 4. Discussion

According to the findings of this study, 59.9% (95% CI: 55, 64.3%) of parents were actively involved in CH services. This study finding is consistent with the study done on Fathers’ involvement in supplementary feeding of infants in Damot Woyde District, South Ethiopia, which found that 50.9% of the fathers had good involvement in CH, such as complementary feeding.^[24]^

This study found higher than a study on fathers’ involvement in child feeding and its associated factors among fathers with children aged 6 to 24 months in Antsokia Gemza Woreda, Ethiopia, which indicated that the level of fathers’ involvement in child feeding was 43.1%.^[25]^

However, this is lower than the study on father involvement in child feeding: perceived responsibility and determinants of participation in Australia, which revealed that they ate meals together frequently/mostly (79%). Many fathers believed that they were responsible for deciding whether their child consumes the “proper kind of foods” (60%),^[26]^ lower than the study conducted on Parenting for Early Childhood Development in Ethiopia, which found that 57% of parents have strong nutritional participation, 78% of children are not left alone, and 70% of children are not put in the care of another kid because of good involvement.^[27]^ Furthermore, the findings lower than a study on fathers’ involvement in breastfeeding practices and associated factors among families having children younger than 6 months in southern Ethiopia revealed that fathers’ involvement in nursing practices was 72.4%.^[20]^ The discrepancy may be due to geographical location, study period, method used, and cultural differences.

Mothers’ level of education, occupation, monthly income, health care service affordability, and knowledge of developmental milestones were all statistically significant predictors of PI in CH care.

When compared to no formal education, moms with elementary and secondary education were 2.82 and 1.76 times more likely to be involved in CH care. This could be because parents with greater education have better health literacy, proactive health behaviors, better communication with healthcare providers, and believe in preventive treatment.

Housewives were 2.08 times more likely than merchants and day laborers to participate in CH services. This could be due to the wife’s more flexible schedule, which allows them to attend medical appointments and follow up on health services, as well as their increased involvement in day-to-day child-rearing activities.

Parents with higher incomes were 3.46 times more likely to participate in CH services than those with lower incomes. Higher-income parents may be able to afford superior health care services, such as private clinics and advanced therapy, encouraging more frequent and proactive involvement in their child’s health. This finding is corroborated by research in Damot Woyde, which found that income was significantly associated with parental participation. Fathers with a household income >500ETB were 56% more involved in their children’s supplemental feeding. Fathers who can provide monetarily are more inclined to interact with and nurture their children.^[24]^

Furthermore, parents who considered health care as affordable were 3.51 times more likely to participate in CH programs. This is due to reduced financial restrictions. Parents who find health care cheap are more likely to seek care early, even for minor health issues, rather than waiting for the condition to develop owing to financial concerns.

Furthermore, parents with good awareness of childhood developmental milestones were 2.33 times more likely to participate in CH services than those with poor knowledge. This could include raising health-care awareness, providing early intervention, and monitoring health at home. This finding is consistent with Damot Woyde’s research, which found that the majority of fathers understand the recommended complementary feeding techniques. Fathers who understand ECD milestones are more likely to participate in child supplemental feeding.^[24]^ In line with a study conducted in Misha woreda, southern Ethiopia, knowledge influences the level of involvement. If dads are aware of the value of nursing, they will provide stronger support to their spouses in breastfeeding and child care activities.^[20]^

## 5. Limitations of the study

This study has several limitations that should be considered when interpreting the findings. First, the cross-sectional design limits the ability to establish causal relationships between PI and ECD-related factors. Second, the study relied on self-reported data, which may be subject to recall bias and social desirability bias, potentially leading to over- or underestimation of PI and related variables.

Third, the findings may have limited generalizability beyond the study area, as the research was conducted in selected woredas of Kolfe Keraniyo Sub City, Addis Ababa, and may not fully represent other urban or rural settings in Ethiopia. Additionally, although a multistage sampling technique was used, there remains a possibility of sampling bias, particularly due to household selection procedures and the exclusion of households without eligible participants at the time of data collection.

Furthermore, parents or caregivers who were critically ill or unable to communicate were excluded from the study, which may have introduced selection bias and limited the representation of more vulnerable groups. Finally, although the questionnaire was adapted from previous studies, the study did not comprehensively report formal reliability and validity measures (such as Cronbach α), which may affect the consistency and reproducibility of the measurements.

## 6. Conclusion

The findings of this study indicate that PI in CH care remains suboptimal among parents of children aged 0 to 59 months in Kolfe Keraniyo Sub City, Addis Ababa. Among the factors examined, mothers’ level of education emerged as the strongest predictor of PI in CH care. Efforts to improve PI should focus on enhancing parental knowledge, particularly regarding early childhood developmental milestones, and improving access to affordable health care services. Interventions should also consider socioeconomic factors, including parental education and household income, to promote equitable engagement in CH care. Future research is recommended to explore these relationships using longitudinal designs to better establish causality.

## Acknowledgments

The authors would like give express their gratitude to the health care professionals at the Hospital for their generosity and invaluable assistance in collecting data and retrieving charts. Moreover, the authors wish to express their gratitude to the data collectors and supervisors.

## Author contributions

**Conceptualization:** Mequanente Dagnaw.

**Data curation:** Mequanente Dagnaw, Ellojita Rout.

**Formal analysis:** Mequanente Dagnaw, Meera Indracanti, Zebiba Kedir, Suleyman Mohammed Arage.

**Funding acquisition:** Mequanente Dagnaw.

**Investigation:** Suleyman Mohammed Arage.

**Methodology:** Meera Indracanti.

## Correction

This article was originally published with an incorrect affiliations “^d^ Department of Public Health, Ayer Tena Health Science and Business College, Addis Abeba, Ethiopia and ^e^ Department of Biochemistry, School of Allied and Healthcare Sciences, Malla Reddy University, Hyderabad, Telangana, India.” These affiliations have now been updated online with “^d^ Department of Microbiology, Malla Reddy Medical College for Women, Malla Reddy Vishwavidyapeeth, Hyderabad, Telangana, India and ^e^ Department of Public Health, Ayer Tena Health Science and Business College, Addis Abeba, Ethiopia.”
